# Mapping insecticide resistance in *Anopheles gambiae* (*s.l.*) from Côte d’Ivoire

**DOI:** 10.1186/s13071-017-2546-1

**Published:** 2018-01-08

**Authors:** Soromane Camara, Alphonsine A. Koffi, Ludovic P. Ahoua Alou, Kouakou Koffi, Jean-Paul K. Kabran, Aboubacar Koné, Mathieu F. Koffi, Raphaël N’Guessan, Cédric Pennetier

**Affiliations:** 1 Institut Pierre Richet/Institut National de Santé Publique (IPR/INSP), BP 1500 Bouake, Côte d’Ivoire; 2 Université Félix Houphouët Boigny (UFHB), 22 BP 582 Abidjan 22, Côte d’Ivoire; 3 London School of Hygiene and Tropical Medicine, London, UK; 4 Institut de Recherche pour le Développement (IRD), Maladies Infectieuses et Vecteurs, Ecologie, Génétique, Evolution et Control (MIVEGEC), UMR 5290 CNRS-IRD-UM, Montpellier, France

**Keywords:** Malaria vectors, Resistance, Insecticides, Côte d’Ivoire

## Abstract

**Background:**

Insecticide resistance in malaria vectors is an increasing threat to vector control tools currently deployed in endemic countries. Resistance management must be an integral part of National Malaria Control Programmes’ (NMCPs) next strategic plans to alleviate the risk of control failure. This obviously will require a clear database on insecticide resistance to support the development of such a plan. The present work gathers original data on insecticide resistance between 2009 and 2015 across Côte d’Ivoire in West Africa.

**Methods:**

Two approaches were adopted to build or update the resistance data in the country. Resistance monitoring was conducted between 2013 and 2015 in 35 sentinel sites across the country using the WHO standard procedure of susceptibility test on adult mosquitoes. Four insecticide families (pyrethroids, organochlorides, carbamates and organophosphates) were tested. In addition to this survey, we also reviewed the literature to assemble existing data on resistance between 2009 and 2015.

**Results:**

High resistance levels to pyrethroids, organochlorides and carbamates were widespread in all study sites whereas some *Anopheles* populations remained susceptible to organophosphates. Three resistance mechanisms were identified, involving high allelic frequencies of *kdr* L1014F mutation (range = 0.46–1), relatively low frequencies of *ace-1*^*R*^ (below 0.5) and elevated activity of insecticide detoxifying enzymes, mainly mixed function oxidases (MFO), esterase and glutathione S-transferase (GST) in almost all study sites.

**Conclusion:**

This detailed map of resistance highlights the urgent need to develop new vector control tools to complement current long-lasting insecticidal nets (LLINs) although it is yet unclear whether these resistance mechanisms will impact malaria transmission control. Researchers, industry, WHO and stakeholders must urgently join forces to develop alternative tools. By then, NMCPs must strive to develop effective tactics or plans to manage resistance keeping in mind country-specific context and feasibility.

## Background

Malaria remains the deadliest tropical infectious disease, causing an estimated 429,000 deaths worldwide in 2015 of which 90% occurred in sub-Saharan Africa [1]. The disease is caused by *Plasmodium* parasites that are transmitted to humans in Africa mainly by *Anopheles gambiae*, *An. coluzzii*, *An. arabiensis* and *An. funestus* [2]. The first three species are members of the *An. gambiae* complex [3]. The dominant mosquito species responsible for the malaria transmission in Côte d’Ivoire are mainly *An. gambiae* and *An. coluzzii* that are widespread over the country [4, 5]. They are well adapted to all types of breeding sites (permanent breeding sites or temporary rain pools such as puddles, shallow wells, footprints, or in rice and vegetable fields) in both rural and urban areas.

Malaria vector control in sub-Saharan Africa relies predominantly on long-lasting insecticidal nets (LLINs) and indoor residual spraying (IRS). The World Health Organization (WHO) recommends different LLINs and IRSs formulations that meet safety and efficacy criteria [6]. The fifteen products recommended for IRS belong to four chemical classes (carbamates, organochlorides, organophosphates and pyrethroids) but only pyrethroid insecticides are currently used in the manufacture of LLINs. However, the widespread use of insecticides for agricultural, domestic or public health purpose has led to the rapid development of insecticide resistance, which constitutes a serious threat to the efficacy of the current arsenal. Resistance to these compounds has been largely described in human disease vectors, especially in the African malaria vectors [7–9].

In 2012, WHO launched a Global Plan for Insecticide Resistance Management in malaria vectors (GPIRM). This plan was widely disseminated to the member states and the African Network on Vector Resistance (ANVR) and served as a springboard to its rollout and implementation in the African region [10]. Subsequently, a roadmap for the implementation of GPIRM was developed by ANVR. Following this roadmap, endemic countries were encouraged to build a strong resistance database and to elaborate a malaria vector control plan for effective management of insecticide resistance in their specific ecological settings. In many countries, resistance data are sparse and not up to date, impeding the design of a solid resistance management plan. The development of a resistance management plan requires a close partnership and collaboration between NMCPs and qualified research institutions to map up resistance in a country.

The current paper presents the results of insecticide resistance monitoring from 2013 to 2015 under the initiative of NMCP. To provide readers with the most exhaustive database, a review of the literature was also carried out for the years 2009 to 2015 and relevant data added. The present document is structured into two main parts: (i) the insecticide resistance phenotypes observed in malaria vector populations sampled across the country, and (ii) the detection of underlying resistance mechanisms.

## Methods

### Study area

**Fig. 1:**
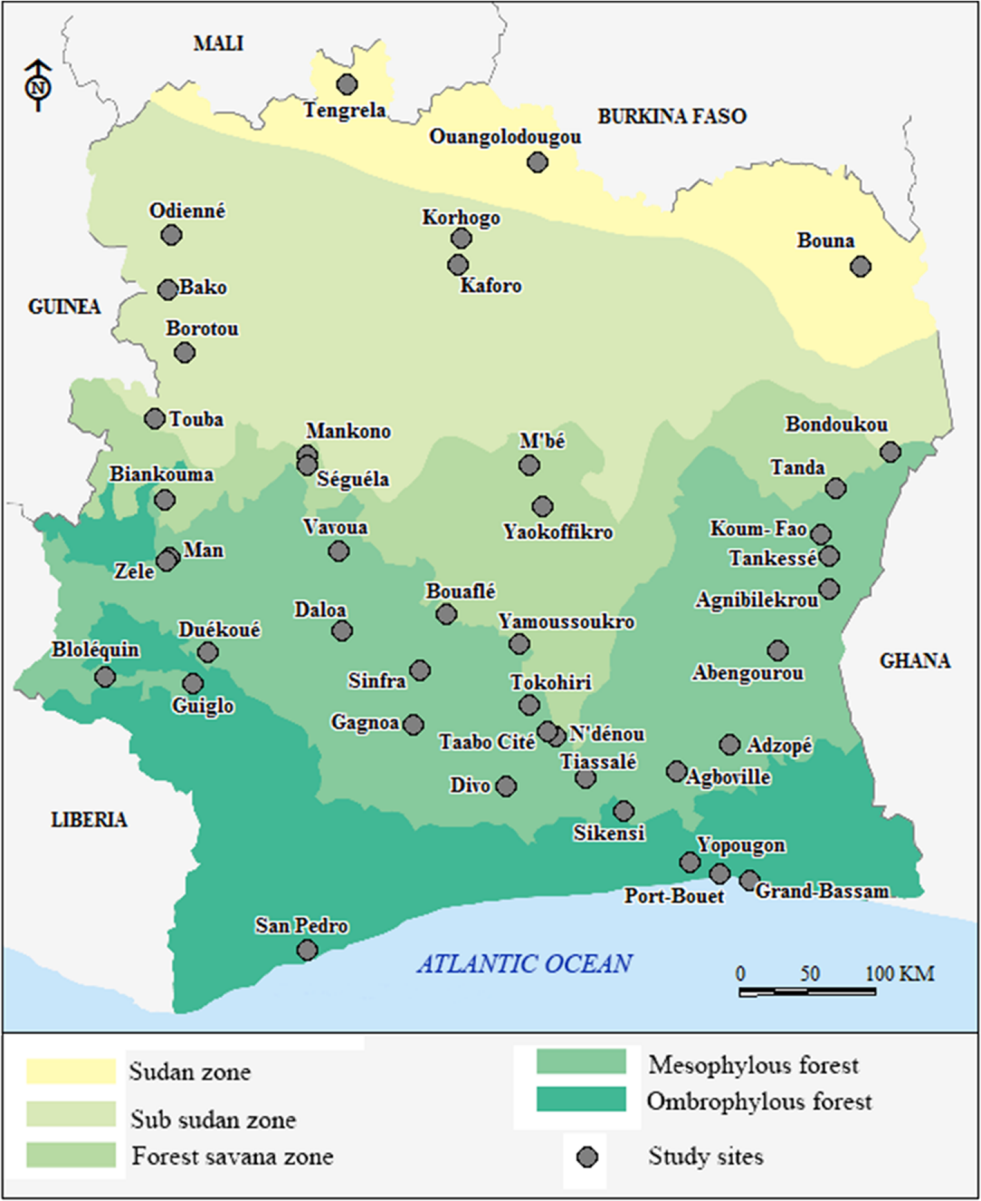
Localization of the *Anopheles* spp. larval collection sites in different ecological zones of Côte d’Ivoire

Côte d’Ivoire is a West African country of 322,462 square kilometres and 22 million inhabitants. It borders Burkina Faso and Mali in the North, Liberia and Guinea in the West, Ghana in the East and the Atlantic Ocean in the South (Fig. 1). The climate is equatorial in the south, tropical in the central region and semi-arid in the far north. Seasons are distinguishable by rainfall and temperature. The country is divided into four climatic zones and seasons characterized by a type of vegetation. Four seasons characterize the Southern region with an equatorial climate: a long rainy season from April to mid-July and a short rainy season from September to November, alternating with a short dry season from mid-July to September and a long dry season from December to March. The climate in the western part is subequatorial, (mountain climate) with similar seasons to the southern region. The south and west part of the country is characterized by dense forest (ombrophile and mesophile forest) whereas the centre has an equatorial climate and opens up to a transition between the south and north. Four seasons are observed: a long rainy season from March to June and a short rainy season from September to October. The long dry season starts in November through to February and a short dry season from July to August. The northern region belongs to the tropical climate (Sudanian climate) with two main seasons: a long rainy season from June to September and a long dry season with rare rainfall from October to May. Vegetation in the northern part of the country consists mainly of the savannah.

### Mosquito collections

*Anopheles* larvae collection were done between June 2013 and December 2015 during the raining season in 35 localities (Fig. 1). To avoid any sampling bias, mosquito larvae were sampled in breeding sites at least one km apart and within a radius of 10 km around each locality. Specimens were then pooled per locality, brought back and reared to the adult stage in the insectary of the Institut Pierre Richet (Bouake, Côte d’Ivoire). Larvae were fed TetraMin® fish food and adults with 10% honey solution.

### Insecticide susceptibility tests

Bioassays were carried out using the standard WHO protocol [11]. Tests were performed with four pyrethroids (0.75% permethrin, 0.05% deltamethrin, 0.05% alpha-cyperméthrin and 0.05% lambdacyhalothrin), one organochloride (4% DDT), two carbamates (0.1% bendiocarb and 0.4% carbosulfan) and three organophosphates (1% fenitrothion, 1% pyrimiphos-methyl and 0.4% chlorpyriphos-methyl). Two to four-days-old unfed adult females of *An*. *gambiae* (*s.l.*) collected at the larval stage and reared until adults emerged were tested. The susceptible Kisumu strain was also tested. Four batches of 25 mosquitoes per insecticide were exposed to the treated papers for 60 min at 25 ± 2 °C and 80% relative humidity. Negative controls consisted of batches of mosquitoes from each site exposed to untreated papers. After exposure, mosquitoes were transferred to the observation tube and provided with 10% honey solution and held for 24 h before scoring mortality. After the bioassays, unexposed mosquitoes (i.e. from the control tubes) were individually dry-frozen at -80 °C for biochemical and molecular analysis.

### Species identification, target site mutations genotyping and biochemical assays

Genomic DNA was extracted from individual mosquitoes using cetyl trimethyl ammonium bromide (CTAB) 2% method described in Yahouedo et al. [12]. Species identification of the *An. gambiae* complex members were done as per Scott et al*.* [13] and Favia et al*.* [14]. The presence of L1014F and L1014S *kdr* mutations were assessed by PCR using the method described by Martinez-Torres et al. [15] and Ranson et al*.* [16]. The PCR-RFLP diagnostic test was used to detect G119S *ace-1*^*R*^ mutation as described by Weill et al*.* [17].

Biochemical assays were performed to quantify the mean activity of mixed function oxidases (MFO), and the mean activities of non-specific esterases (NSE) for alpha- and beta-naphthyl acetate and glutathione S-transferases (GST) using female mosquitoes unexposed as described by WHO [18].

### Other data sources

In addition to the 35 localities where bioassays were carried out, data from 8 localities spread over the country were obtained *via* PubMed, research centres and national universities, leading to a total of 43 localities investigated for insecticide resistance in malaria vectors. A literature search of published papers and reports from national institutions on insecticide resistance in the country using WHO standard assays was performed. We used Côte d’Ivoire, *Anopheles*, *An. gambiae*, insecticide resistance, *kdr* and *Ace-1*^*R*^ as keywords in database searches.

The literature search was done on PubMed and Google Scholar during December 2015. Data were also extracted from reports of national universities and research institutes. After careful reading, the following data were recorded: locality name, GPS coordinates (longitude and latitude) of locality and mosquito collection period. For each site, entomological data were extracted and added to the insecticide resistance database generated with WHO tube test results. These data were species of *Anopheles*, insecticide class/type/dosage, number of mosquitoes exposed per assay, mortality rates and susceptibility status. Regarding the resistance mechanisms, data extracted were the frequency of target site mutations, detoxifying enzyme quantities and/or activity rates together with the analysis protocols that had been used.

### Data analysis

Insecticides susceptibility or resistance status was determined following WHO criteria. According to WHO recommendation made in 2013, 98–100 mosquitoes’ mortality indicate susceptibility, 90–97% suggests a suspected resistance that needs to be confirmed, < 90% mortality indicate resistance. Genotypic frequencies at *kdr* L1014F and *ace-1*^*R*^ G119S loci in *An. gambiae* (*s.l.*) wild populations were tested for goodness-of-fit to Hardy-Weinberg expectations using Fischer's exact tests generated with the Genepop 4.0 software [19]. Biochemical assay data (enzymatic activity per mg protein) were compared between a susceptible *An. gambiae* reference strain (Kisumu) and the wild vector populations from twenty-two sites by a Kruskal-Wallis non-parametric test.

## Results

### Insecticide susceptibility across the country

The mortality rate of the Kisumu reference strain to all insecticides was 100% indicating the good bio-availability of the insecticide active ingredients on the papers.

Insecticide susceptibility status of the wild population of *An. gambiae* (*s.l.*) was tested across Cote d’Ivoire with pyrethroid insecticides in 43 localities. Pyrethroid resistance was already widespread in all study sites (Table 1). Nevertheless, populations from two areas in the northern part (Kaforo and Korhogo) appeared to be fully susceptible to deltamethrin (98–100% mortality). There were still some appreciable levels of mortality with this insecticide among populations from the east side (Adzope and Abengourou) (91–97% mortality) and one place in the centre (Tokohiri) (92%), although they fall within WHO range for suspicion of resistance. Similar suspicion of resistance to permethrin was noticed in those from Man, Ndenou, Taabo Cite and Yopougon (93–98% mortality).

**Table 1:** Pyrethroid and organochlorine susceptibility of wild population of *Anopheles gambiae* (*s.l.*) in 43 sites in Côte d’Ivoire

Locality	Pyrethroids												Organochlorine			
	Permethrin 0.75%			Deltamethrin 0.05%			Alphacypermethrin 0.05%			Lambdacyhalothrin 0.05%			DDT 4 %			
	*n*	% Mort	Status	*n*	% Mort	Status	n	% Mort	Status	*n*	% Mort	Status	*n*	% Mort	Status	Ref
Abengourou	101	61.8	R	101	97.0	RS	105	82.9	R	–	–	–	106	66.1	R	
Adzopé	100	42.0	R	96	90.6	RS	–	–	–	102	67.7	R	–	–	–	[54]
Agboville	–	–	–	116	62.9	R	–	–	–	–	–	–	–	–	–	[55]
Agnibilekro	99	23.2	R	103	74.3	R	103	20.4	R	–	–	–	101	0.3	R	
Bako	–	–	–	108	0.9	R	–	–	–	–	–	–	–	–	–	
Biankouma	100	0	R	97	28.9	R	101	9.9	R	–	–	–	–	–	–	
Bloléquin	20	10.0	R	100	6.0	R	–	–	–	–	–	–	–	–	–	
Bondoukou	105	17.1	R	104	50.9	R	104	9.6	R	101	37.6	R	98	8	R	
Borotou	70	5.7	R	103	16.5	R	101	9.9	R	–	–	–	–	–	–	
Bouaflé	99	13.1	R	70	54.3	R	–	–	–	96	9.4	R	106	0.9	R	
Bouna	83	30.9	R	–	–	–	–	–	–	–	–	–	–	–	–	
Daloa	105	18.1	R	102	43.1	R	98	24.5	R	99	7.1	R	–	–	–	
Divo	–	–	–	99	64.6	R	–	–	–	–	–	–	–	–	–	[55]
Duékoué	103	2.9	R	105	12.4	R	–	–	–	101	4.9	R	–	–	–	
Gagnoa	103	6.8	R	98	33.7	R	73	2.7	R	–	–	–	–	–	–	
Grand-Bassam	202	2.5	R	178	37.1	R	202	11.4	R	108	3.7	R	103	0.9	R	
Guiglo	100	1.0	R	102	5.9	R	68	7.3	R	–	–	–	–	–	–	
Kaforo	–	–	–	51	100	S	–	–	–	–	–	–	–	–	–	
Korhogo	97	31.1	R	101	98.1	S	100	70.0	R	–	–	–	105	6.5	R	
Koum- Fao	102	40.2	R	–	–	–	–	–	–	–	–	–	–	–	–	
Man	94	93.6	RS	88	73.9	R	99	73.8	R	104	10.6	R	101	40.6	R	
Mankono	–	–	–	73	39.7	R	–	–	–	–	–	–	–	–	–	
M'bé	201	51.1	R	274	75.8	R	240	67.9	R	–	–	–	193	3.1	R	
N'dénou	103	93.0	RS	103	85.0	R	–	–	–	–	–	–	108	5.5	R	[56]
Odiénné	98	5.1	R	106	8.5	R	105	5.7	R	–	–	–	–	–	–	
Ouangolo	104	53.4	R	107	59.5	R	99	42.4	R	102	68.3	R	98	2.5	–	
Port-Bouet	98	50.0	R	103	54.4	R	97	75.3	R	101	16.8	R	102	35.3	R	
San pedro	106	73.0	R	125	78.7	R	106	7.5	R	106	11.6	R	75	58.5	R	
Séguela	102	5.9	R	98	29.6	R	99	21.2	R	–	–	–	–	–	–	
Sikensi	–	–	–	81	51.9	R	–	–	–	–	–	–	–	–	–	[55]
Sinfra	104	0	R	101	7.9	R	99	2.0	R	–	–	–	–	–	–	
Taabo cité	99	96.9	RS	107	89.7	R	–	–	–	–	–	–	111	17.1	R	[56]
Tanda	86	66.3	R	89	69.6	R	79	69.6	R	108	30.6	R	80	37.6	R	
Tankéssé	108	9.2	R	102	35.3	R	104	8.7	R	101	10.1	R	106	1.3	R	
Tengrela	104	21.8	R	104	41.3	R	104	8.7	R	104	15.4	R	104	15.4	R	
Tiassalé	204	24.0	R	282	31.9	R	–	–	–	–	–	–	306	8.2	R	[24]
Tokohiri	103	54.4	R	107	91.6	RS	–	–	–	–	–	–	105	0	R	[56]
Touba	101	1.9	R	106	62.3	R	100	28.0	R	–	–	–	–	–	–	
Vavoua	108	11.1	R	99	7.1	R	104	17.3	–	–	–	–	–	–	–	
Yamoussoukro	125	73.7	R	102	23.5	R	100	50.0	R	100	16.8	R	101	3.0	R	
Yaokoffikro	106	68.9	R	101	11.9	R	105	5.7	R	97	68.0	R	99	1.0	R	
Yopougon	98	92.2	RS	104	89.4	R	99	100	S	–	–	–	102	29.4	R	
Zele	77	74.2	R	–	69.2	R	104	82.7	R	–	–	–	93	43.4	R	

*Abbreviations*: *n*, number of tested mosquitoes; –, no value; % *Mort*, mortality rate 24 h post-exposure; *S*, susceptibility; *RS*, suspected resistance; *R*, resistance; *Ref*, reference

DDT resistance was widespread, with mortality ranging between 0–43% in 21/23 areas tested (Table 1). There was resistance to the carbamates (carbosuflfan and bendiocarb) except in Duekoue in the west and Sans Pedro in the south where resistance to bendiocarb was not clear-cut and therefore under suspicion.

Organophosphate insecticides (chlorpyrifos-methyl, pyrimifos-methyl and fenithrotion) induced far higher mortality than any other insecticide family. Indeed, populations from all areas except Tiassale showed full susceptibility (99–100% mortality) to chlorpyrifos-methyl. With pyrimifos-methyl, nearly half of the areas tested (11/21) were fully susceptible and the over half resistant or suspected for resistance, with various levels of mortality scored in the bioassays (4–83% for confirmed resistance and 91–96% for the suspicion of resistance) (Table 2).

**Table 2:** Organophosphate and carbamate susceptibility of wild population of *Anopheles gambiae* (*s.l.*) in Côte d’Ivoire

Locality	Carbamate						Organophosphates									
	Bendiocarb 0.1 %			Carbosulfan 0.4%			Fenitrothion 1%			Pyrimifos-metyl 1%			Chlorpyrifos-methyl 0.4 %			
	*n*	% Mort	Status	*n*	% Mort	Status	*n*	% Mort	Status	*n*	% Mort	Status	*n*	% Mort	Status	Ref
Abengourou	–	–	–	101	14.3	R	–	–	–	100	100	S	–	–	–	
Agboville	116	69.0	R	–	–	–	103	62.1	R	94	16.0	R	–	–	–	[55]
Agnibilekro	101	67.3	R	–	–	–	–	–	–	–	–		99	100	S	
Biankouma	103	57.3	R	–	–	–	100	82.0	R	98	100	S	–	–	–	
Bondoukou	106	89.6	R	–	–	–	–	–	–	–	–	–	104	100	S	
Bouaflé	99	48.5	R	–	–	–	106	77.4	R	101	72.3	R	–	–	–	
Daloa	93	67.7	R	–	–	–	103	94.2	RS	91	91.2	RS	–	–	–	
Divo	199	77.9	R	–	–	–	196	98.0	S	58	43.8	R	–	–	–	[55]
Duékoué	103	91.3	RS	–	–	–	103	99.0	s	98	100	S	–	–	–	
Gagnoa	–	–	–	–	–	–	103	98.1	S	–	–	–	–	–	–	
Grand-Bassam	124	52.4	R	–	–	–	102	83.0	R	108	3.7	R	103	99.0	S	
Guiglo	107	85.0	R	–	–	–	98	100	S	100	100	S	–	–	–	
Kaforo	–	–	–	–	–	–	–	–	–	–	–	–	–	–	–	
Korhogo	–	–	–	97	14.8	R	–	–	–	99	82.7	R	–	–	–	
Man	–	–	–	88	52.0	R	–	–	–	101	99.0	S	–	–	–	
Mankono	–	–	–	–	–	–	–	–	–	–	–	–	–	–	–	
M'bé	99	65.7	R	200	6.5	R	102	99.0	S	102	100	S	100	100	S	
Odiénné	99	32.3	R	–	–	–	82	67.3	R	–	–	–	–	–	–	
Ouangolodougou	107	59.8	R	–	–	R	–	–	–	–	–	–	104	100	S	
Port-Bouet	102	9.8	R	–	–	R	99	96.0	RS	101	70.4	R	–	–	–	
San pedro	105	92.4	RS	97	35.9	R	–	–	–	–	–	–	79	100	S	
Séguela	97	23.7	R	–	–	–	97	68.0	R	100	100	S	–	–	–	
Sikensi	108	56.5	R	–	–	–	111	82.2	R	–	–	–	–	–	–	[55]
Sinfra	99	68.7	R	–	–	–	106	96.2	RS	100	100	S	–	–	–	
Tanda	106	83.0	R	–	–	–	–	–	–	–	–	–	101	100	S	
Tankéssé	105	80.0	R	–	–	–	76	93.4	RS	–	–	–	76	100	S	
Tengrela	103	72.8	R	–	–	–	101	93.1	RS	–	–	–	99	100	S	
Tiassalé	209	12.4	R	217	2.8	R	296	74.0	R	100	68.0	R	192	82.8	R	[24]
Touba	100	51.0	R	–	–	–	102	83.3	R	98	100	S	–	–	–	
Vavoua	101	68.3	R	–	–	–	104	96.2	RS	96	100	S	–	–	–	
Yamoussoukro	97	52.6	R	104	42.7	R	100	91.0	RS	100	96.3	RS	–	–	–	
Yaokoffikro	100	42.0	R	79	13.9	R	83	91.6	RS	102	100	S	99	100	S	
Yopougon	–	–	–	100	25.0	R	–	–	–	98	63.3	R	–	–	–	

*Abbreviations*: *n*, number of tested mosquitoes; –, no value; % *Mort*, mortality rate 24 h post-exposure; *S*, susceptibility; *RS*, suspected resistance; *R*, resistance; *Ref*, reference

### Species and target site mutation distribution

Species identification showed that *Anopheles gambiae* (*s.l.*) samples were composed of two species; *Anopheles gambiae* (*s.s*.) and *Anopheles coluzzi. Anopheles gambiae* was dominant in the sub-Sudanian area whereas *An. coluzzi* was dominant in the forest area.

The L1014F and L1014S *kdr* mutations were studied in 29/43 sites investigated. There was no L1014S *kdr* mutation found in *An. gambiae* (*s.s*.) samples but the presence of the L1014F mutation was observed in all populations from the twenty-nine sites. Allelic frequencies varied from 0.4 to 1. Most of the *An. gambiae* (*s.s*.) populations (18/29) displayed high frequencies (*kdr* frequency ≥ 0.8) whereas, moderate allelic frequencies between 0.5–0.8 were detected in eight populations. The allelic frequencies were below 0.5 in only 3 out of 29 populations tested (Table 3). The *ace-1*^*R*^ G119S mutation was detected in 25 areas analyzed. Tiassale and Yaokoffikro populations had the highest allelic frequencies (*ace-1*^*R*^ frequencies = 0.50). The frequencies in the other areas ranged between 0–0.35 (Table 3).

**Table 3:** Frequency of the *kdr* L1014F and *ace-1*^*R*^ G119S mutations in *An. gambiae* (*s.l.*) from Côte d’Ivoire

Locality	*An. coluzzi*				*An. gambiae* Giles				Frequency mutation in *An gambiae* (*s.l.*) population			Ref
	*kdr* L1014F		*ace-1*^*R*^ G119S		*kdr* L1014F		*ace-1*^*R*^ G119S					
	*n* ^*a*^	F (*kdr*)	*n* ^*a*^	F (*ace-1*^*R*^)	*n* ^*a*^	F (*kdr*)	*n* ^*a*^	F (*ace-1*^*R*^)	*n* ^*b*^	F (*kdr*)	F (*ace-1*^*R*^)	
Abengourou	29	0.517	29	0	29	0.666	3	0	32	0.531	0	
Adzopé	–	–	–	–	–	–	–	–	90	0.67	–	[54]
Agnibilekro	5	1	5	0.200	39	1	39	0.269	44	1	0.261	
Bingerville	21	0.881	20	0.225	11	0.682	11	0.409	32	0.813	0.290	
Bondoukou	6	1	6	0.083	40	1	40	0.113	46	1	0.109	
Bouna	12	1	12	0.292	24	1	24	0.125	36	1	0.181	
Daloa	2	1	2	0.5	44	0.966	44	0.261	46	0.967	0.272	
Divo	–	–	–	–	–	–	–	–	–	0.807	–	
Grand-Bassam	36	0.986	41	0.329	4	1	5	0.200	40	0.988	0.315	
Kaforo	10	0.800	39	0.389	20	0.825	21	0.238	31	0.817	0.283	
Korhogo	9	0.722	8	0.062	23	0.761	23	0.283	32	0.750	0.226	
Koum-Fao	6	1	6	0.167	34	0.971	33	0.136	40	0.975	0.141	
Man	–	–	–	–	–	–	–	–	60	0.840	0.020	
M'bé (Bouaké)	–	–	–	–	–	–	–	–	226	0.800	0.020	
N'Denou	–	–	–	–	–	–	–	–	32	0.558	–	[56]
Ouangolodougou	5	1	5	0.500	45	1	45	0.033	50	1	0.315	
Port-bouet	31	0.855	32	0.359	0	–	0	–	32	0.855	0.359	
San-pedro	46	1	46	0.174	0	–	0	–	46	1	0.174	
Sassandra	45	0.856	45	0.054	0	–	0	–	45	0.856	0.054	
Taabo cité	–	–	–	–	–	–	–	–	32	0.611	–	[56]
Tanda	1	1	1	0.500	45	0.935	45	0.267	46	0.978	0.272	
Tankéssé	15	1	15	0.067	31	0.935	30	0.033	46	0.957	0.044	
Tengrela	22	0.955	22	0.136	24	1	24	0.208	46	0.978	0.174	
Tiassalé	–	–	–	–	–	–	–	–	–	0.830	0.500	[24]
Tokohiri	–	–	–	–	–	–	–	–	32	0.519	–	[56]
Yamoussoukro	26	0.479	26	0.135	5	0.500	5	0.100	31	0.484	0.125	
Yaokoffikro	–	–	–	–	–	–	–	–	111	0.940	0.500	
Yopougon	31	0.468	32	0.172	–	–	–	–	32	0.468	0.172	
Zele	7	0.5	7	0	25	0.500	25	0.260	32	0.500	0.203	

^a^Number of mosquitoes tested by species

^b^Total number tested

*Abbreviations*: F(*kdr*), frequency of the kdr mutation; F(*ace-1*^*R*^), frequency of the *ace-1*^*R*^ mutation

### Levels of enzyme activity

The quantity or activity of three main detoxifying enzyme families was measured in 22 vector populations from Côte d’Ivoire. Elevated alpha- esterase was observed in 16 populations and beta-esterase in 12 sites, in comparison to susceptible mosquitoes. The highest esterase activity was recorded for *An. gambiae* (*s.l.*) from Yopougon, Port-Bouët, Korhogo and Yaokoffikro (Kruskal-Wallis H-test: *χ*^2^ = 64.60, *df* = 21, *P* < 0.001) (Table 4). Oxidase activity was more elevated in nine populations than in Kisumu mosquitoes (Kruskal-Wallis H-test: *χ*^2^ = 67.74, *df* = 22, *P* < 0.001). The highest levels of oxidase activity were observed in Kaforo in the North and Man in the West, with 4-fold higher activity than in Kisumu (Table 4). Elevated GST activity was observed in 90% (18/20) of the populations relative to activity in the Kisumu mosquitoes (Kruskal-Wallis H-test: *χ*^2^ = 61.78, *df* = 20, *P* < 0.001). The highest GST activity (> 4-fold) was recorded for *An. gambiae* (*s.l.*) from Yopougon, Korhogo, Yamoussoukro and Port-Bouët (Table 4).

**Table 4:** Mean level of detoxifying enzyme activity in *Anopheles gambiae* (*s.l.*) in Côte d’Ivoire

Locality	Alpha-esterase	Beta-esterase	Oxidases (MFO)	Glutathione S-transferase (GST)
	μmol α-naphtol/min/mg protein (*n*)	μmol β-naphtol/min/mg protein (*n*)	Nmol P450/mg protein (*n*)	Nmol GST conj/min/mg protein (*n*)
Kisumu	0.086 (40)	0.084 (40)	0.095 (38)	0.295 (40)
Abengourou	0.036 (32)	0.043 (32)	0.251* (31)	0.501(25)
Kaforo	0.155* (15)	0.105 (15)	0.330* (15)	–
Korhogo	0.340* (32)	0.206* (32)	0.209* (23)	1.236* (21)
Man	0.150* (32)	0.175* (32)	0.420* (31)	0.664* (26)
Port-bouet	0.300 * (17)	0.365* (15)	0.236* (12)	1.065* (14)
Yamoussoukro	0.192* (32)	0.138* (33)	0.198* (32)	1.008* (32)
Yaokoffikro	0.336* (113)	–	0.039 (113)	0.154 (108)
Yopougon	0.352* (32)	0.313* (32)	0.255* (32)	2.358* (24)
Zele	0.107 (32)	0.089 (32)	0.163 (33)	0.638* (28)
Agnibilekro	0.158* (51)	–	0.104 (52)	0.412* (45)
Bondoukou	0.159* (43)	0.198* (37)	0.115 (43)	0.389* (27)
Bouna	–	0.094 (23)	0.071 (33)	–
Daloa	0.052 (43)	–	0.077 (42)	0.639* (42)
Grand-Bassam	0.133* (50)	0.109 (43)	0.164* (50)	0.471* (44)
Koum-Fao	0.144* (46)	0.158* (49)	0.066 (55)	0.470* (52)
M'bé (Bouaké)	0.155* (40)	0.125* (40)	0.198* (36)	0.378* (40)
Ouangolodougou	0.127 (48)	0.105 (33)	0.079 (43)	0.362* (40)
San-pedro	0.079 (50)	0.148* (50)	0.034 (50)	0.356* (41)
Sassandra	0.136* (35)	0.089 (30)	0.066 (35)	0.805* (35)
Tanda	0.204* (45)	0.230* (48)	0.094 (49)	0.380* (41)
Tankéssé	0.183* (53)	0.137* (53)	0.133* (53)	0.515*(53)
Tengrela	0.173* (58)	0.169* (56)	0.065 (54)	0.356*(41)

*Note*: Kisumu result expresses enzyme activity in susceptible reference strain

*Abbreviations*: n, number of mosquitoes tested; –, value not determined

*P < 0.05: Enzyme level significantly higher compared to Kisumu strain

## Discussion

A better management of insecticide resistance in malaria vectors relies on detailed and frequent resistance monitoring. Other vector bionomic data are also essential to build a relevant strategic plan. Local capacity with technical skills and financial support need to be strengthened to create the best environment to achieve such monitoring. In Côte d’Ivoire, the NMCP is working in close collaboration with universities and research institutions to build on the best evidence-based strategic plan to fight malaria. As a contribution to this approach, we updated the insecticide resistance status among malaria vectors in Côte d’Ivoire, relying on recent data package generated between 2009 and 2015. This study focused on *An. gambiae* (*s.l.*), the major malaria vector in the country despite the presence of secondary vectors such as *An. funestus* and *An. niili* are also involved in malaria transmission. Overall, resistance to pyrethroids, organochlorides and carbamates is general in malaria vector populations of Côte d’Ivoire. Resistance to organophosphate insecticides was also widespread, but some malarial mosquito populations were still susceptible whereas some displayed moderate levels of resistance.

National research institutions [20] recently (2012) showed susceptibility to pyrethroids and organochlorides in the West and V-Baoulé in the central country. Moreover, all vector populations tested with fenitrothion and chlorpyrifos-methyl (organophosphates) were susceptible before 2009 [20]. The widespread and strong resistance both to organochlorides and pyrethroids as well as the moderate level of resistance to organophosphates in the present study illustrates the recent rapid evolution and spread of insecticide resistance to almost all public health insecticides. The distribution of resistance was patchy, and its intensity varies from one malaria vector population to another, highlighting the local selection of such phenotypes [21] as already illustrated in *Culex pipiens* species [22].

This rapid evolution relies on the selective pressure that has multiple origins. It was shown that agricultural use of pesticides and public health and domestic use of insecticide contribute to the selection and spread of resistance mechanisms in malaria vector populations in Côte d’Ivoire. The cause-consequence link is very difficult to establish; nevertheless, several papers suggested a link between the emergence and spread of the *kdr* and *ace-1*^*R*^ mutations in *An. gambiae* (*s.s*.) populations of Côte d’Ivoire and agricultural use of pyrethroids, carbamates and organophosphates insecticides to protect cotton, cocoa, coffee and rice crops [5, 23, 24]. In urban areas, another selection pressure source suggested is the pesticides used for vegetable growing [25]. Some studies have also shown that mosquito larvae exposed to sub-lethal doses of pollutants, herbicides or pesticides display an insecticide resistance through increased activities of insecticide-detoxifying enzymes [26, 27].

The highest frequencies of the *kdr* mutation have been observed in the north which is a cotton growing area, in the east, in Abidjan, in San Pedro, Tiassalé, Daloa and in the Bouake area. The overall allele frequency of *ace-1*^*R*^ was higher in Tiassalé and Yaokoffikro, both rice-growing areas. These mutations have also been detected with high frequencies in *An*. *gambiae* (*s.l.*) in many regions of West Africa [28, 29] with ecological similarities in Burkina Faso and Benin (cotton, rice and urban/sub-urban vegetable growing areas) [30, 31].

Currently, the increasing use of insecticides for malaria vector control plays an important role in the insecticide selection pressure. According to Mathias et al. [32], ITN coverage for populations has been associated with a development of strong insecticide selection pressure by pyrethroid and increase of *kdr* allele frequency. A reduction in the susceptibility of *An. gambiae* (*s.l.*) populations subjected to ITNs was also observed in Uganda [33]. Besides that, increase in *kdr* frequencies was evidenced in *An. gambiae* (*s.l.*) after the distribution of LLINs in Kenya [34], Niger [35], Senegal [36] and Benin [37]. In Côte d’Ivoire, more than 10,000,000 LLINs were distributed in 2014. This might have contributed to the selection pressure of insecticide resistance.

This study has also shown the involvement of detoxification enzymes in insecticide resistance in Côte d’Ivoire. The underlying metabolic mechanisms are currently under the spotlight, and a growing number of studies are investigating the cellular and molecular mechanisms that are driving such resistance phenotypes.

Despite the multiple resistance observed in the country, LLINs effectiveness has, until now, never been questioned [38]. However, some studies have shown a decrease of entomological efficiency in strong resistance zone [39]. Such LLINs insecticidal efficacy decrease has also been recently observed in the M’bé station, in central Côte d’Ivoire (Ahoua Alou et al. submitted) where multiple resistance was confirmed a few years ago [40].

In this context, operational research urgencies are obvious. First of all, it is essential to decipher the interaction between insecticide resistance and operational efficacy or failure. In 2009, the WHO Global Malaria Programme with funding from the Bill & Melinda Gates Foundation initiated a multi-country study to ascertain the potential loss of epidemiological effectiveness of these core insecticidal interventions as a result of decreased susceptibility of malaria vectors to the insecticides used [41]. The longitudinal study was completed in four African countries (Benin, Cameroon, Kenya and Sudan) in 2016 and found no association between resistance and malaria prevalence where LLIN use was high [42].

Alternatives are currently available or under development. These alternatives could be classified into two main groups. The first comprises technologies that improve the efficacy of existing class of insecticide treated tools. In this group belong new LLINs treated with a synergist such as piperonyl butoxide and pyrethroids insecticide. Some of these have shown promising results against wild free-flying multi-resistant *An. gambiae* (*s.s*.) in experimental huts [34, 43–45]. This technology recommended by WHO aims to alleviate levels of resistance by removing the metabolic component of resistance due to MFOs and NSEs. The second group represents a group of new LLINs under development combining two insecticides with a different mode of action seems to be promising for controlling resistant mosquitoes [46]. This second category gathers complementary tools that could complement the massive distribution of LLINs (i.e. indoor residual spraying, larviciding, outdoor vector control set up and use of drugs with insecticidal properties such as Ivermectin) [47, 48]. Nevertheless despite the proof of principle of their potential efficacy, the randomized control trials showing the benefit to use them at community scale are scarce [49], and their ecological scope of the application does not seem to be global. Updated insecticide resistance data will also help to choose relevant areas to implement such randomized controlled trials [50, 51].

The NMCP of Côte d’Ivoire plans to integrate IRS and larviciding into malaria control policies. Given the high resistance to carbamates, pyrethroids and organochloride all over the country, our results indicate that only organophosphates (such as pyrimiphos-methyl or chlorpyriphos-methyl) could be advisable for IRS deployment.

Finally, physiological resistance mechanisms are closely interacting with behavioural adaptations in malaria vectors. There is an increasing number of evidence that malaria vector populations behave differently after wide insecticide pressure, due to a large LLINs distribution. These behavioural changes may affect the effectiveness of LLINs or IRS by reducing the possibility for a mosquito to contact insecticides in multiple ways as reviewed in Gatton et al*.* [52]*.* Behavioural adaptations and evolution of both mosquito and human populations are of crucial interest if the scientific community aims to understand better the residual transmission. As an example, a recent study highlighted the long-range effect (deterrent or attractive) of ITNs that may have significant consequences for personal and community protection against malaria transmission [53].

## Conclusion

This study showed a wide distribution of pyrethroids, organochlorides and carbamate resistance in *An. gambiae* (*s.l.*) in Côte d’Ivoire. Resistance to organophosphates was also widespread but some vector populations were still susceptible whereas others displayed a moderate level of resistance. These results could be used as a basis for the development of a strategic framework for strengthening malaria vector control implementation and insecticides resistance management. The rapid evolution since the last decade and patchy distribution of such resistance phenotypes indicate that monitoring insecticide resistance is essential and must be considered by authorities for the implementation of malaria control programs in Côte d’Ivoire.

## Abbreviations

NMCP: National Malaria Control Programmes
WHO: World Health Organization
LLINs: Long-lasting insecticidal nets
IRS: Indoor residual spraying
L1014F *kdr*: West knockdown resistance
*ace-1*^*R*^: Acetylcholinesterase-1 resistance
NSE: Non-specific esterase
MFO: Mixed-function oxidase
GST: Glutathione S-transferase
GPIRM: Global Plan for Insecticide Resistance Management
ANVR: African Network on Vector Resistance
IPR: Institut Pierre Richet
*ace-1*^*R*^ G119S: G199S mutation in *ace-1*^*R*^
DDT: Dichlorodiphenyltrichloroethane
R: Resistant
S: Susceptible
